# AGE AS A FACTOR IN DIVERSITY, EQUITY, AND INCLUSION INITIATIVES IN HIGHER EDUCATION

**DOI:** 10.1093/geroni/igad104.3167

**Published:** 2023-12-21

**Authors:** Natalie Galucia, Nancy Morrow-Howell

**Affiliations:** Washington University in St. Louis, St. Louis, Missouri, United States; Washington University in St. Louis, St. Louis, Missouri, United States

## Abstract

At institutions of higher education, demographic shifts in the population and the growth of diversity, equity, and inclusion (DEI) initiatives are occurring simultaneously. This study seeks to understand their intersection by focusing on age as a diversity factor in DEI initiatives on college campuses. Six focus groups with a total of 42 DEI staff members were convened, including 36 colleges/universities across the country. Thematic data analysis was conducted by three members of the research team who reached consensus on themes, subthemes, and representative quotes. Focus group participants suggest that age as an identity factor is not given much attention in DEI initiatives. Participants acknowledge the issue of age; but in general, they strive to keep other identities, like race and gender, in the forefront, especially in the face of low resources. These findings can be understood in the context of the current political environment around social justice issues and the pervasive ageism at the society level, especially in age-segregated institutions of higher education. While it may be difficult to elevate age in DEI initiatives on campuses, interventions were identified; and advocacy efforts may move us toward advancing age diversity, equity, and inclusion in higher education as well as other organizations.

